# Species‐ and sex‐dependent changes in body size between 1892 and 2017, and recent biochemical signatures in rural and urban populations of two ground beetle species

**DOI:** 10.1002/ece3.10329

**Published:** 2023-07-21

**Authors:** Silvia Keinath, Johannes Frisch, Johannes Müller, Frieder Mayer, Ulrich Struck, Mark‐Oliver Rödel

**Affiliations:** ^1^ Museum für Naturkunde, Berlin – Leibniz Institute for Evolution and Biodiversity Science Berlin Germany; ^2^ Berlin‐Brandenburg Institute of Advanced Biodiversity Research – BBIB Berlin Germany; ^3^ Department of Earth Science Freie Universität Berlin Berlin Germany

**Keywords:** carbon, *Harpalus affinis*, *Harpalus rufipes*, nitrogen, spatio‐temporal gradient, stable isotopes

## Abstract

Increasing urbanisation and intensified agriculture lead to rapid transitions of ecosystems. Species that persist throughout rapid transitions may respond to environmental changes across space and/or time, for instance by altering morphological and/or biochemical traits. We used natural history museum specimens, covering the Anthropocene epoch, to obtain long‐term data combined with recent samples. We tested whether rural and urban populations of two ground beetle species, *Harpalus affinis* and *H. rufipes*, exhibit spatio‐temporal intraspecific differences in body size. On a spatial scale, we tested signatures of nitrogen and carbon stable isotopes enrichments in different tissues and body components in recent populations of both species from urban and agricultural habitats. For body size examinations, we used beetles, collected from the early 20th century until 2017 in the Berlin‐Brandenburg region, Germany, where urbanisation and agriculture have intensified throughout the last century. For stable isotope examinations, we used recent beetles from urban and agricultural habitats. Our results revealed no spatio‐temporal changes in body size in both species' females. Body size of *H. rufipes* males decreased in the city but remained constant in rural areas over time. We discuss our findings with respect to habitat quality, urban heat and interspecific differences in activity pattern. Although nitrogen isotope ratios were mostly higher in specimens from agricultural habitats, some urban beetles reached equal enrichments. Carbon signatures of both species did not differ between habitats, detecting no differences in energy sources. Our results indicate that increasing urbanisation and intensified agriculture are influencing species' morphology and/or biochemistry. However, changes may be species‐ and sex‐specific.

## Graphical abstract

In our study, we examined spatio‐temporal morphological and biochemical (stable isotopes) trait changes in two ground beetle species from urban and rural populations. Our results show that increasing urbanisation across the past 125 years, and intensified agriculture affect therein living species. However, trait changes might be species‐ and sex‐specific.

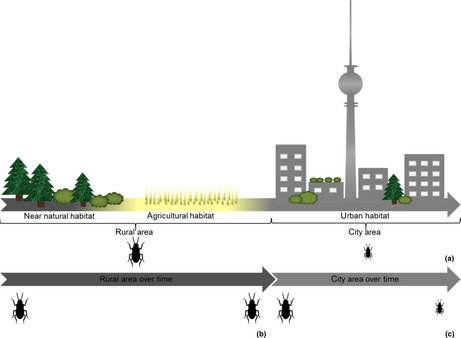

## 1 INTRODUCTION

The human demand for land, mostly for settlements and agriculture, is increasing globally (Antrop, 2004; Bairoch & Goertz, 1986). In urban areas, exhaust gases from industry and traffic produce pollution (Fenger, 1999), artificial night lighting leads to an imbalance between day and night (Falchi, 2019), and construction works conduce fragmentation and loss of habitats (Liu et al., 2016). The increasing proportion of artificial surfaces, for example for buildings and roads, is influencing microclimatic conditions, resulting in increasing temperatures of 1–5°C in cities compared to undeveloped surrounding areas (Kalnay & Cai, 2003). Such urban heat islands (Oke, 1973; Rizwan et al., 2008) will be more accentuated by global warming (Alcoforado & Andrade, 2008), resulting in more frequent heat waves in cities (Fenner et al., 2018). In agricultural landscapes, however, monocultures lead to habitat homogenisation (Jongman, 2002), the use of herbicides and/or insecticide influence biodiversity negatively (Geiger et al., 2010) and the application of chemical and/or biological fertilisers result into increased nitrogen enrichments, as well as other nutrients, to the environment (Freyer & Aly, 1974; Jenkinson, 2001; Shearer et al., 1974; Vitousek et al., 1997; Zarzycki & Kopeć, 2020). These environmental changes can cause to rapid transitions to altered environments over time (Hobbs et al., 2013; Jeltsch et al., 2013), influencing species composition and function, giving rise to anthropogenically impacted ecosystems (Harris et al., 2006; Ricciardi, 2007; Root & Schneider, 2006). Consequently, many native species disappear from anthropogenically impacted ecosystems, whereas non‐native species establish via human transport or range extension (Kenis et al., 2009; Ziegler, 2011). However, some native species persist through the transition process (Doudna & Danielson, 2015; Van't Hof et al., 2011), because they are able to cope with or adapt to new conditions (Cook & Saccheri, 2013; Giraudeau et al., 2014).

In the Berlin‐Brandenburg region, Germany, rapid transitions to anthropogenically impacted environments took place across time, resulting in a densely populated metropolis of Berlin, surrounded by the rural federal state of Brandenburg that is mainly covered by agricultural monocultures (Cochrane & Jonas, 1999). For answering the question on how native species manage to persist through such rapid transitions to anthropogenically impacted environments, natural history museum collections can provide a source of such persisting species, covering time series of one region. For this purpose, morphological traits of museum voucher specimens can be used as proxies for species' ecological and physiological modifications over time (Keinath et al., 2020, 2021; Niemeier et al., 2020; Rocha et al., 2014). These data sets can be complemented with recent samples to cover spatio‐temporal gradients (Doudna & Danielson, 2015; Keinath et al., 2020, 2021; Niemeier et al., 2020; Van't Hof et al., 2011).

Body size is a useful morphological trait, indicating habitat quality (Blake et al., 1994; Chungu et al., 2018; Kotze & O'Hara, 2003; Lövei & Magura, 2022; Magura et al., 2020; Niemelä et al., 2002), and correlates with many life‐history aspects, such as reproduction rate, dispersal ability or developmental time (Peters, 1983). Gray's increasing disturbance hypothesis predicts that mean body size should decrease from less to more disturbed habitats, in the present context from rural to urban areas (Gray, 1989).

**FIGURE 1:**
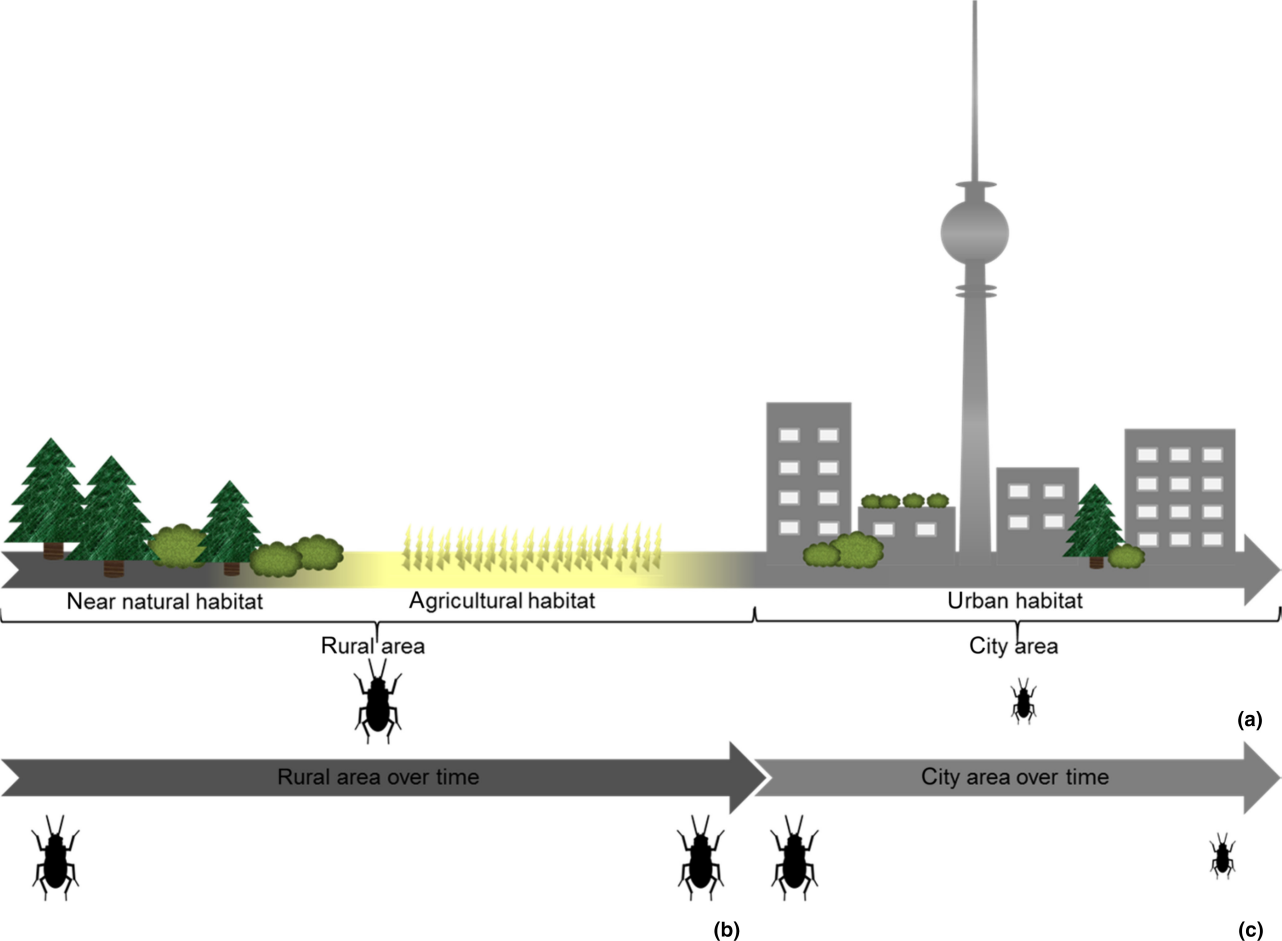
Predicted anthropogenic influence on body size of ground beetles, in space and time. Along a land‐use gradient we expected to find smaller‐sized beetles in urban habitats compared to agricultural habitats (a), in rural areas (composed of near‐natural and agricultural habitats) we expected body sizes to change to a lesser extent (b), whereas we expected beetles to shrink in the city over time (c).

Especially ectothermic animals, such as insects, may be impacted by urbanisation because their development depends on environmental temperature, and thus might be sensitive to changing microclimatic conditions and anthropogenically modified habitats in cities. Is the food source of an insect larva limited, higher temperatures lead to faster larval development and an earlier start of metamorphosis, resulting in smaller‐sized imagines (Gillooly et al., 2001; Kingsolver & Huey, 2008). In holometabolic insects, this mechanism is driven by the timing of key hormonal events involved in moulting and metamorphosis (Davidowitz et al., 2003, 2004). Thus, living in the city may result in smaller‐sized insects in comparison with insects occurring in cooler rural surroundings (Brans et al., 2017; Kotze et al., 2011) when rural habitat quality is more suitable than the urban one (Gillooly et al., 2001; Kingsolver & Huey, 2008; Figure 1).

Apart from morphological traits stable isotope composition reflects the habitat conditions of an individual (Peterson & Fry, 1987; Tieszen & Boutton, 1988). In organisms, carbon and nitrogen are enriched with two stable isotopes, ^13^C and ^12^C, and ^15^N and ^14^N, respectively (Rosing et al., 1998). The stable carbon isotope signatures provide information about the respective energy base of an animal's diet (Gratton & Forbes, 2006; Ponsard & Arditi, 2000), reflecting the relative proportion of C3 and C4 plants in its respective environment (Degens, 1969; Schwarcz, 1969). The nitrogen isotope composition provides information about the position of an individual in the food web (Birkhofer et al., 2011), and whether the individual lived in a nitrogen rich environment, such as in an agricultural landscape with intense application of chemical and/or biological fertilisers (Freyer & Aly, 1974; Jenkinson, 2001; Shearer et al., 1974; Vitousek et al., 1997). Thus, populations showing different ^15^N/^14^N ratios may indicate different habitat conditions in which the populations are occurring, such as in intensively managed agricultural and less impacted habitats (Birkhofer et al., 2011).

The composition of stable isotopes may also differ in different tissues and/or body components, depending on its respective turnover time. Consequently, it is possible to determine long‐term as well as short‐term dietary preferences of one individual specimen. Chitin has slow turnover and because holometabolic insects are not moulting after metamorphosis, its isotopic signature reflects the nutritive status of an individuals' larval stage. However, tissues with fast turnover, like muscles, reflect the dietary conditions of an adult individual (Gratton & Forbes, 2006; Peterson & Fry, 1987). Thus, analyses of stable isotopes from different tissues and/or body components may provide information about whether different development stages of an individual exploit nutrient sources from different habitats that differ in their nitrogen or carbon enrichments. Different stable isotope enrichments may thus indicate that an individual dispersed from one into another habitat (Hood‐Nowotny & Knols, 2007; Schallhart et al., 2009).

Among holometabolic insects, carabid beetles are a suitable and well‐known indicator group of human‐caused disturbances and habitat quality, such as urbanisation and agricultural land use. They are common in most terrestrial habitats (Chungu et al., 2018; Duan et al., 2019; Koivula, 2011; Lövei & Magura, 2022; Lövei & Sunderland, 1996; Magura & Lövei, 2021; Martinson & Raupp, 2013; Piano et al., 2020; Sukhodolskaya et al., 2021; Sustek, 1992; Weller & Ganzhorn, 2003).

Ground beetles respond differently to urbanisation (Magura et al., 2020, 2021; Sadler et al., 2006; Sukhodolskaya & Saveliev, 2014). On a community level, Blake et al. (1994) and Ribera et al. (2001) showed that smaller ground beetle species are more abundant in highly disturbed areas than larger ones, whereas Magura et al. (2020) mainly found smaller species of silvicolous ground beetles in urban areas, due to their higher sensitiveness to habitat fragmentation and higher environmental temperatures. On an intraspecific level, the studies by Weller and Ganzhorn (2003) and Weller (2000) showed decreasing body size in the generalist ground beetle species *Carabus nemoralis* (Müller, 1764) towards the city centre.

For our study, we selected two ground beetle species, *Harpalus affinis* (Schrank, 1781) and *H. rufipes* (De Geer, 1774). Both species are common and well‐represented in the collection of the Museum für Naturkunde, Berlin, comprising specimens from Berlin and Brandenburg that cover the past 125 years and are easy to collect in recent times in order to extend time series and for stable isotope examinations. Both species occur in little impacted environments and in open, altered habitats such as meadows, pastures, arable land and urban fallows and parks (Anjum‐Zubair et al., 2015; Deichsel, 2006; Harrison & Gallandt, 2012; Sunderland et al., 1995; Townsend, 1992). Although both species are generalists in adult and larvae stages, they predominantly feed on weed seeds (Bažok et al., 2007; Luff, 2002; Sunderland et al., 1995; Toft & Bilde, 2002; Townsend, 1992; Trautner, 2017).

The first aim of our study is to examine changes in body sizes in urban and rural populations of *Harpalus affinis* and *H. rufipes* across the last 125 years in the Berlin‐Brandenburg area, Germany. Reasoned by both beetles' predominantly food source, weed seeds, we predicted urban habitats to be lower in habitat quality than rural landscapes with high amount of agriculture. With additionally higher temperatures in urban areas, we assumed specimens of both species to be smaller when occurring in urban areas, whereas we assumed body size in specimens of both species occurring in rural areas to exhibit overall larger body size. We further predicted both species to be smaller in recent times than in the past, due to increasing habitat destructions and temperatures in the city of Berlin over time, whereas we assumed both species' body size to decrease to a lesser degree in our rural study region over time reasoned by higher habitat quality for both species and lower temperature increase (Brans et al., 2017; Kotze et al., 2011; Li et al., 2018; Figure 1). Additionally, we anticipate higher variability in body size in both species when occurring in the city, due to higher habitat heterogeneity in urban areas (such as parks, ruderal areas, private gardens, green strips) than in rural landscapes (Falk, 1976; Gill & Bonnett, 1973; Tischler, 1973).

Second, we aim to examine stable nitrogen and carbon isotope enrichments in different tissues and body components of both study species in recent populations from intensely managed agricultural habitats and urban habitats. We predicted higher nitrogen enrichment in beetles occurring in agricultural habitats, compared to urban ones, reflecting the use of fertilisers as shown in Birkhofer et al. (2011) for agricultural arthropods. As a consequence of different plant assemblages, we assumed stable carbon isotope composition to differ between beetles originating from agricultural versus urban habitats (Ponsard & Arditi, 2000). Finally, we expected intraspecific differences of isotopic signatures in different tissues and/or body components (Gratton & Forbes, 2006; Peterson & Fry, 1987), if adult beetles dispersed into habitats that differ in nitrogen enrichments to those of the larval stage (Hood‐Nowotny & Knols, 2007; Schallhart et al., 2009).

## 2 MATERIALS AND METHODS

### 2.1 Study area

**FIGURE 2:**
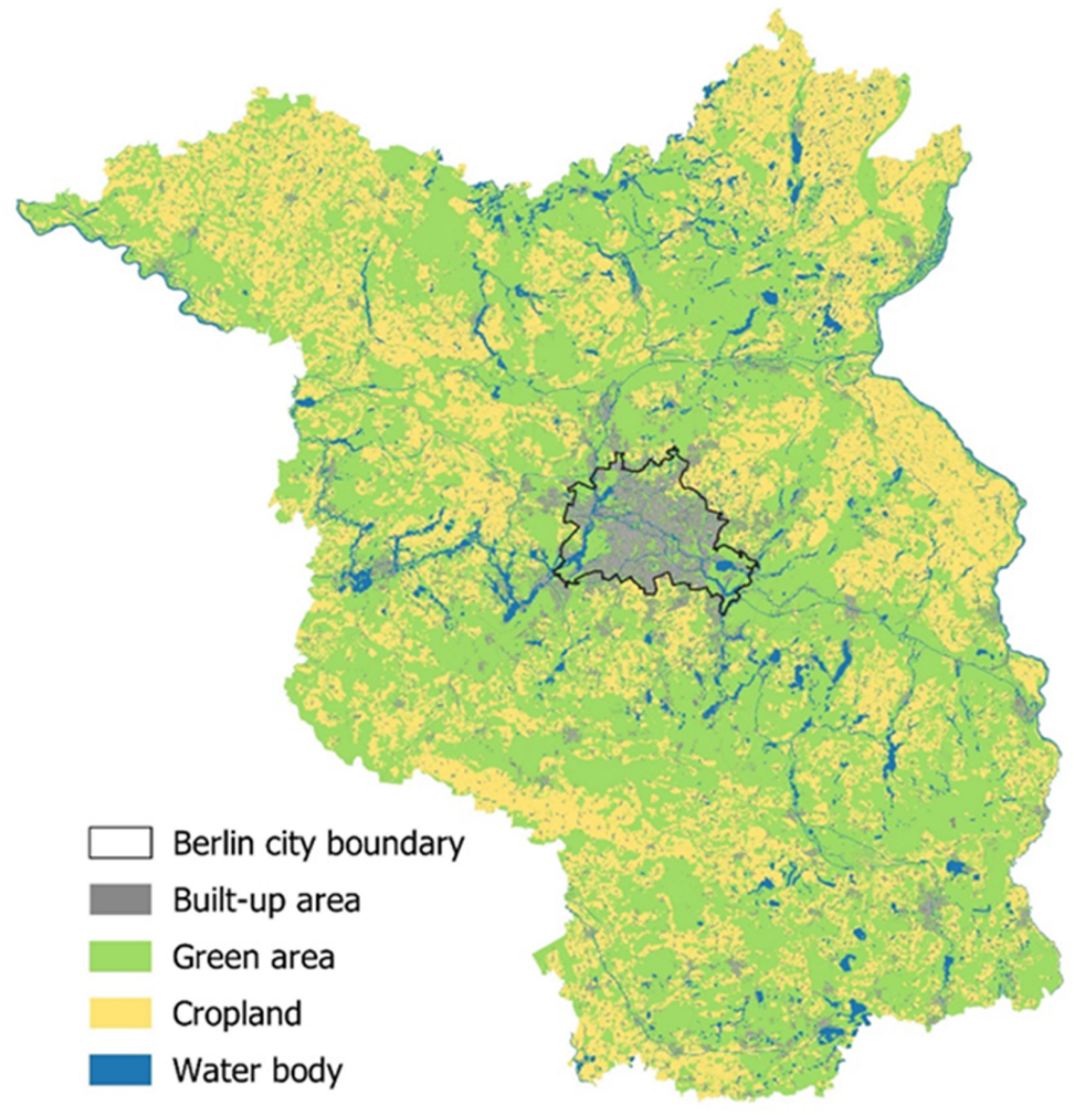
Land‐use map of the Berlin‐Brandenburg area, Germany (data: 2009 to 2015). The rural federal state of Brandenburg, mostly consisting of agricultural and near‐natural landscapes, is surrounding the metropolis of Berlin (Landesamt für Umwelt, 2009; Senate Department for Urban Development and Housing, 2014; Maps merged and prepared by Anne Hiller).

In the Berlin‐Brandenburg region of Germany, rapid environmental transitions occurred especially during the last century (Cochrane & Jonas, 1999). The city of Berlin is a fast‐growing metropolis with increasingly high level of urbanisation and an increasing human population (Antrop, 2000). Brandenburg, the German federal state surrounding Berlin, mostly consists of rural areas comprising near‐natural environments, as well as intensively managed agricultural monocultures (Cochrane & Jonas, 1999; Figure 2). In 2017, Berlin comprised 4108 inhabitants/km^2^, whereas only 84 inhabitants/km^2^ lived in Brandenburg (Statistik Berlin Brandenburg, 2007). Li et al. (2018) measured higher temperatures in Berlin compared to its rural surroundings, that is 5.38 and 2.84 K between day and night‐time, respectively. By using long‐term data (1893–2017), Fenner et al. (2018) found a significant temperature increase in Berlin, including increasing frequencies of summer heat waves.

### 2.2 Study species

The ground beetle (Coleoptera, Carabidae) *Harpalus affinis* is widespread in Europe (Wrase, 2004), and introduced in Canada and New Zealand (Sunderland et al., 1995; Townsend, 1992). It is a medium‐sized (8.5–12 mm), diurnal generalist of open habitats, predominantly feeding on weed seeds and occasionally on insect larvae (Sunderland et al., 1995; Townsend, 1992) and has a variable metallic colouration (Keinath et al., 2020; Wrase, 2004). Adults are winged and volant (Townsend, 1992; Trautner, 2017) and are most active during the breeding period in May and June (Trautner, 2017).

*Harpalus rufipes* is widespread in the Palaearctic, medium‐sized (11–16 mm), predominantly nocturnal (Wrase, 2004) and attracted to artificial light sources (Kegel, 1990; Matalin, 2003; Szentkirályi et al., 2003). Adults and larvae are generalists in deforested habitats and tend to be granivorous (Bažok et al., 2007; Luff, 2002; Toft & Bilde, 2002; Trautner, 2017). The species is most active during the breeding period between July and August (Trautner, 2017).

Both species are generalists of open environments across a wide range of different habitats from arable fields (Harrison & Gallandt, 2012; Sunderland et al., 1995), vineyards, grasslands (Trautner, 2017) to semi‐natural (Anjum‐Zubair et al., 2015; Horák, 2011) and less human impacted landscapes (Holec et al., 2006; Townsend, 1992) as well as in urban green spaces (Deichsel, 2006). Both species tend to be pest species in agricultural fields (Briggs, 1957, 1965). Males differ from females by wider tarsi of the pro‐ and mesothoracal legs (Lindorth, 1945; Townsend, 1992).

### 2.3 Data classification

We examined 562 and 86 museum vouchers of *Harpalus affinis* and *H. rufipes*, respectively, collected between 1892 and 1998, from the Berlin‐Brandenburg region, Germany. From the same region, we examined 62 *H. affinis* and 94 *H. rufipes* specimens, collected in May to July 2016–2017. Berlin museum vouchers of both species with available habitat information have been collected at roadsides, parks, dumpsites, garden plots and ruderal sites. Museum vouchers from Brandenburg were collected in meadows, forest edges and arable landscapes. Recent specimens from Berlin of both species have been sampled at eight urban dry grassland sites, whereas specimens from Brandenburg originated from agricultural winter wheat and soy fields and from fallow grass stripes in between fields, collected in the administrative districts Nordwestuckermark, Uckermark and Märkisch‐Oderland. Beetles collected in recent times (2016–2017) were taken from pitfall trapping from cooperative research projects (see a summary of classifications and numbers of specimens in Table 1; all data are given in https://doi.org/10.7479/stwb‐nf68).

**TABLE 1:** Numbers of examined specimens of *Harpalus rufipes* and *H. affinis* per sex, city and rural areas, time period (1 = 1892–1949; 2 = 1957–1998; 3 = 2016–2017) and urban and agricultural habitats that could be clearly assigned from museum specimens labels or from recent fieldwork.

Numb. Of specimens	***Harpalus rufipes***												***Harpalus affinis***											
	178												624											
Thereof numb. per sex	**Male**						**Female**						**Male**						**Female**					
	95						83						382						242					
Thereof numb. per area	**City**			**Rural**			**City**			**Rural**			**City**			**Rural**			**City**			**Rural**		
	40			55			21			62			183			199			118			124		
Thereof numb. per time period	**1**	**2**	**3**	**1**	**2**	**3**	**1**	**2**	**3**	**1**	**2**	**3**	**1**	**2**	**3**	**1**	**2**	**3**	**1**	**2**	**3**	**1**	**2**	**3**
	20	9	11	16	9	30	9	5	7	10	6	46	48	115	20	50	130	19	32	75	11	39	73	12
Numb. clearly defined habitats from the respective area (city; rural)	**Urban**			**Agricultural**			**Urban**			**Agricultural**			**Urban**			**Agricultural**			**Urban**			**Agricultural**		
	15			30			9			46			23			19			12			12		

All beetles were classified by species, sex and origin. For body size analyses, beetles from Berlin were classified as originating from ‘city area’, Brandenburg specimens were classified as originating from ‘rural area’. Beetles from recent fieldwork and museum vouchers with detailed habitat information were used for in depth habitat comparisons between ‘urban habitats’ and ‘agricultural habitats’. Therefore, we classified beetles from ‘city areas’ into ‘urban habitats’ when sampled in ruderal sites, dumpsites, edges of streets and parks, and beetles from ‘rural areas’ into ‘agricultural habitats’ when sampled in agricultural landscapes (see numbers of examined beetles per classification in Table 1). We suspect that museum vouchers of both species without any further sampling information except ‘Berlin’, collected in the 19th century, were sampled at the periphery of Berlin. However, we assigned them to the category ‘city area’ because the periphery of Berlin, even at that time, can be already regarded as urbanised in comparison with its rural surroundings (Reif, 2002).

For temporal comparisons, we included beetles classified as originating from ‘city area’ and ‘rural area’. Beetles of each category were assigned to one of three distinct time periods: 1892–1949, 1957–1998 and 2016–2017. Time period classifications were based on a combination of practical reasons (availability of sufficient numbers of beetles) and the history of urbanisation and agricultural techniques in our study region (see Keinath et al., 2020).

During the first time period (1892–1949), the Berlin‐Brandenburg region was mainly impacted by the effects of the industrial revolution and First and Second World Wars (Ribbe et al., 2002a, 2002b). Berlin expanded to ‘Groß‐Berlin’ in the 1920s, resulting in rapid construction of buildings and streets (Buesch & Haus, 1987). Then, the Berlin population was larger than ever (Ribbe et al., 2002b). Industrialisation led to high level of environmental pollution (Pamme, 2003). During the second time period (1957–1998), the reconstruction of Berlin was finalised. Berlins' human population was, however, much below the pre‐war conditions (Ribbe et al., 2002b; Schildt & Sywotlek, 1993). By the end of the 1950s, the first environmental protection measures were implemented, including efforts to reduce environmental pollution in the city (Pamme, 2003; UBA, 1998; UNEP/WHO, 1993). Therefore, we treated this time period as an intermediate stage of environmental destruction and pollution. From 1999 to 2017, the Berlin population continuously increased (United Nations, 2018), and environmental protection measures became politically established (Pamme, 2003). Whereas numerous museum specimens were available from the first two time periods, only a few specimens were available from the most recent period. We therefore complemented the museum specimens by additional sampling (see all data https://doi.org/10.7479/stwb‐nf68).

### 2.4 Body size measurements

Body size was measured with the aid of a dissecting microscope and a measuring ocular (Leica MS5), using a revolving table placed under the microscope to enable planar measurements of the pinned beetles. Standardised body length (SBL) measurements, following Kavanaugh (1979), comprised head length (distance from labium to vertex behind eyes), pronotal (maximal distance from anterior to posterior margin) and elytral length (sutural length; distance from posterior end of the scutellum to posterior end of the elytra along suture). Measurement errors (*H. affinis*: ±0.06 mm; *H. rufipes*: ±0.09 mm) were determined by the mean accuracy of repeated measurements of a randomly chosen subset of 10 specimens per species. All measurements were taken by one person without prior knowledge of sex, region of origin and habitat of the respective specimens.

### 2.5 Stable nitrogen and carbon isotopic composition

For stable nitrogen and carbon isotope analyses, we selected 20 recently collected specimens of each species. We examined 10 specimens (5 males and 5 females) of *H. affinis* from nine dry grassland sites in Berlin, and the same number of specimens of *H. rufipes* from seven dry grassland sites in Berlin. Furthermore, we examined 10 specimens of each species (each 5 males and 5 females) from agricultural winter wheat fields and adjacent green strips from Nordwestuckermark (see all data https://doi.org/10.7479/stwb‐nf68).

From all individuals, we removed and weighed 1 mg of legs, cuticula of elytra, and thorax muscle tissues, respectively. The samples were dried in an oven at 40°C for 12 h. Stable isotope analysis and concentration measurements of nitrogen and carbon of the respective samples were performed simultaneously with a THERMO Fisher Scientific Delta V isotope ratio mass spectrometer, coupled to a THERMO Flash EA 1112 elemental analyser via a THERMO Conflo IV‐interface, in the stable isotope laboratory of the Museum für Naturkunde, Berlin. Stable isotope ratios are expressed in the conventional delta notation (δ^13^C/δ^15^N) relative to atmospheric nitrogen (Mariotti, 1983) and VPDB (Vienna PeeDee Belemnite standard). Standard deviation for repeated measurements of lab standard material (peptone) was generally lower than 0.15‰ for nitrogen and carbon, respectively. Standard deviations of concentration measurements of replicates of our lab standard were <3% of the concentration analysed. From the *H. affinis* muscle samples, seven (2 agricultural; 5 urban individuals) comprised insufficient material for analyses and were excluded.

### 2.6 Statistical analyses

We used the Program R, version 3.4.0 (R Core Team, 2017) for all analyses. For testing normal distributions, we used Shapiro Wilk tests. As our data were non‐normally distributed, and other statistical methods we tested (GLM for Poisson distribution and Permanova) were not suitable for our data, we used non‐parametric Wilcoxon rank sum test for comparing standardised body lengths (SBL) between sexes (females, males) ‘SBL ~ Sex’; areas (rural, city) ‘SBL ~ Area’ and habitats, (urban, agricultural) ‘SBL ~ Habitat’. For comparing SBL between the three time periods (1892–1949; 1957–1998; 2016–2017) for each area (rural, city), we used the non‐parametric Kruskal–Wallis rank sum test, ‘SBL ~ Time‐Period’, and Dunn test for single comparisons between the respective time periods with Holm's sequential Bonferroni correction for multiple testing (Dunn. test: Dinno, 2017; FSA‐package: Ogle et al., 2019), based on three tests. We further used Levene's test for testing variation of SBL between habitats, areas and time periods, the latter by using Holm's sequential Bonferroni correction for multiple testing, based on three tests.

For analyses of stable nitrogen (δ^15^N) and carbon (δ^13^C) isotopes comparisons of different tissues and body components (cuticula, legs, thoracic muscles), between sexes ‘δ^15^N ~ Sex’; ‘δ^13^C ~ Sex’ and between agricultural and urban habitats ‘δ^15^N ~ Habitat’; ‘δ^13^C ~ Habitat’, we used the non‐parametric Wilcoxon Rank Sum test. For comparing δ^15^N and δ^13^C isotopic enrichments between tissues and body components either of agricultural or urban habitats ‘δ^15^N ~ Tissue’; ‘δ^13^C ~ Tissue’, we applied the Kruskal–Wallis Rank Sum tests. We further used Levene's test to test variation of stable nitrogen and carbon isotopes of different tissues and body components within the respective habitat and species, both by using Holm's sequential Bonferroni correction for multiple testing, based on each three tests.

## 3 RESULTS

### 3.1 Body size

**FIGURE 3:**
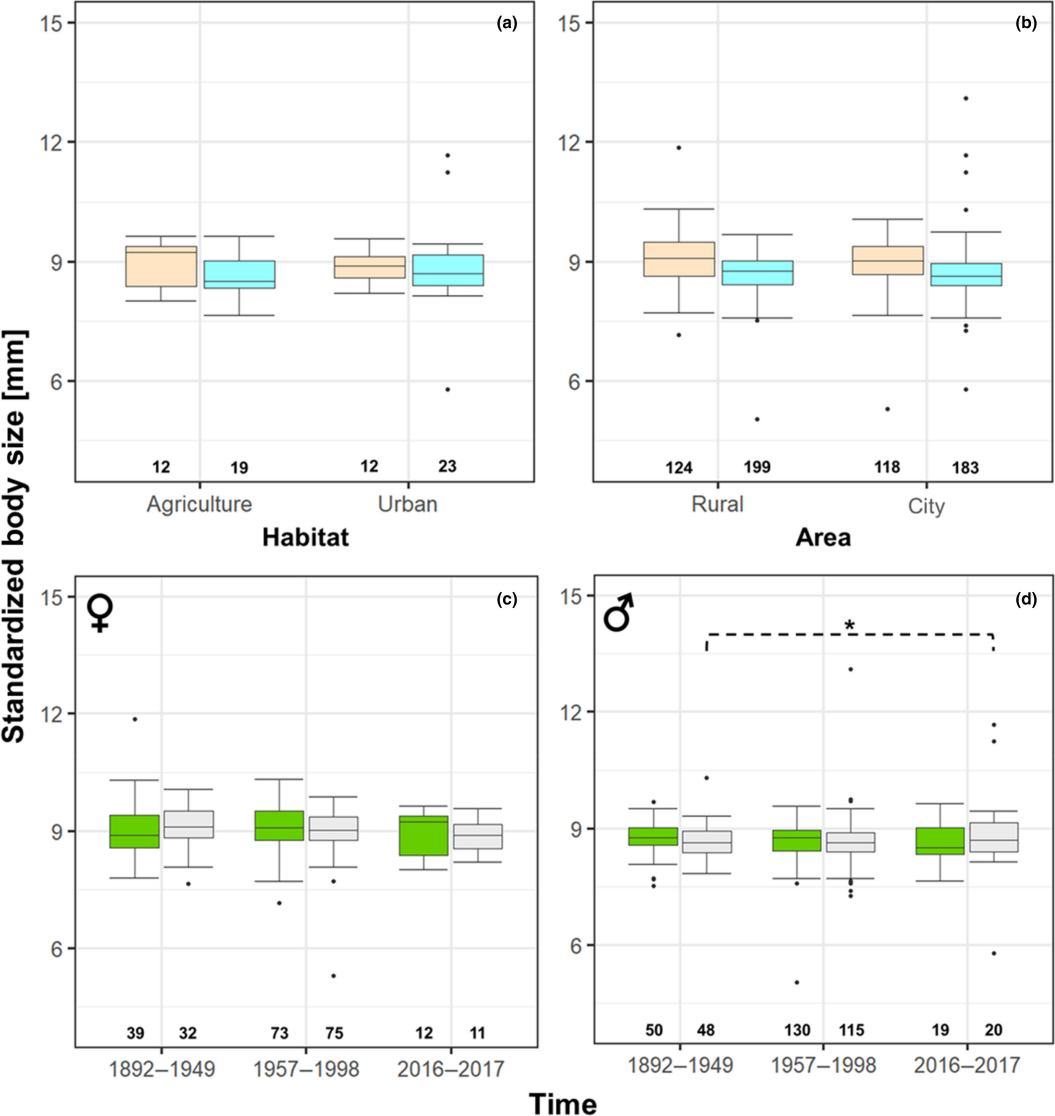
Body size of *Harpalus affinis* from agricultural and urban habitats (a) and rural and city areas (b) of females (rose boxes) and males (blue boxes); females (c) and males (d) in rural (green boxes) and city (grey boxes) areas over time. Boxes mark the interquartile ranges, whiskers indicate the minimum and maximum values, horizontal lines in boxes indicate medians, dots above and below boxes represent outliers, numbers below boxes indicate sample sizes, dotted brackets with stars indicate significant differences in variability of body size (*p < .05).

**FIGURE 4:**
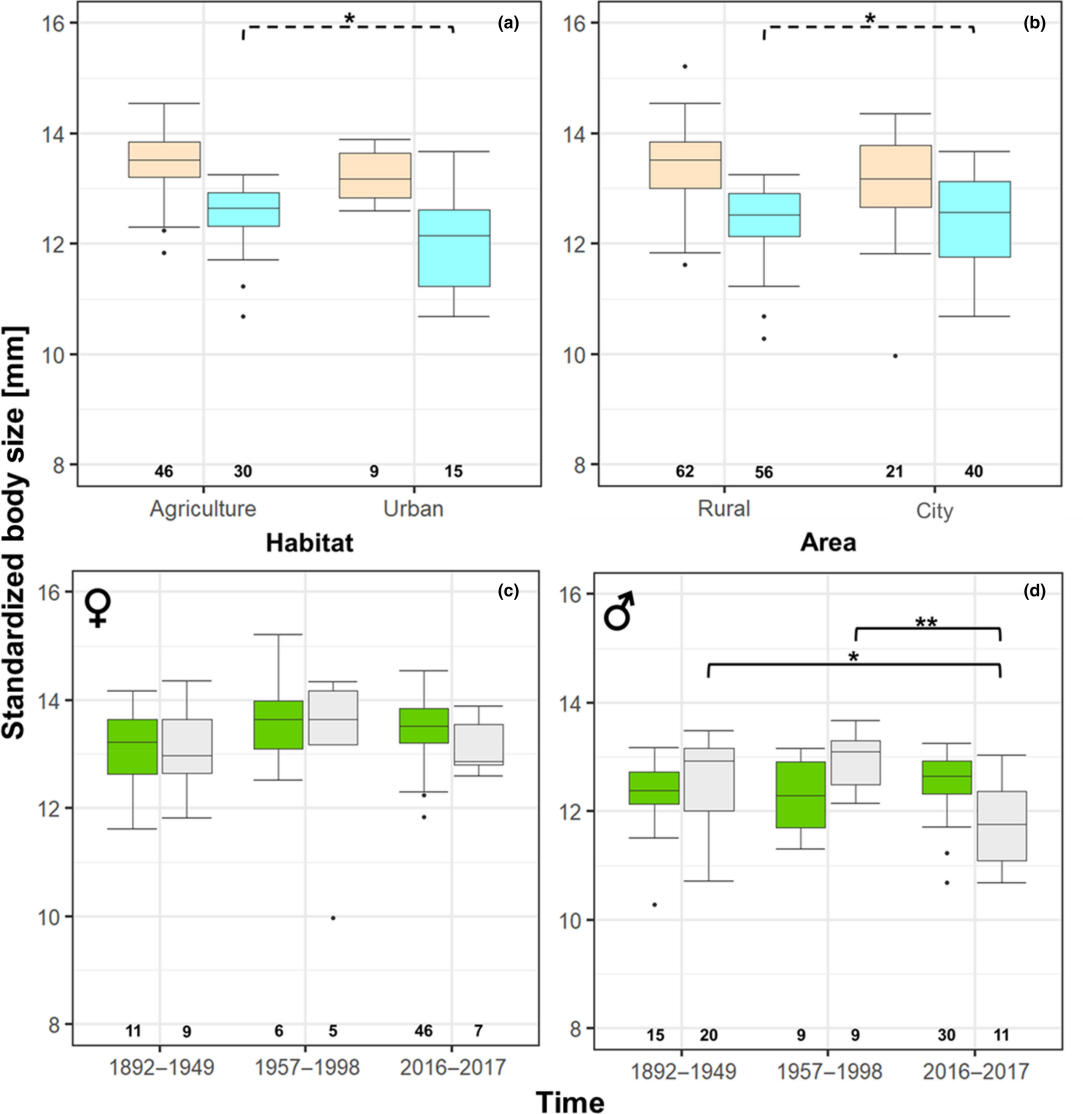
Body size of *Harpalus rufipes* from agricultural and urban habitats (a) and rural and city areas (b) of females (rose boxes) and males (blue boxes); females (c) and males (d) in rural (green boxes) and city (grey boxes) areas over time. Boxes mark the interquartile ranges, whiskers indicate the minimum and maximum values, horizontal lines in boxes indicate medians, dots above and below boxes represent outliers, numbers below boxes indicate sample sizes, dotted brackets with stars indicate significant differences in variability of body size, drawn through brackets with stars indicate significant differences in body size (*p < .05; **p < .01).

Females were significantly larger than males in both species (*H. affinis*: *N* = 624; *p* < .001; females: median 9.01 ± SE 0.04 mm; males: median: 8.70 ± SE 0.03 mm; *H. rufipes*: *N* = 178; *p* < .001; females: median: 13.46 ± SE 0.09 mm; males: median: 12.53 ± SE 0.08 mm; Tables 2 and A1 in Appendix 1). Neither species in both sexes differed in their body size between habitats (agricultural vs. urban) nor between areas (rural vs. city; Tables 2 and A1; Figures 3 and 4a,b). Likewise, body size in females in both species remained constant in rural and city areas over time (Tables 2 and A1; Figures 3c and 4c). This was also the case for male *H. affinis* (Tables 2 and A1; Figure 3d) from rural habitats over time.

**TABLE 2:** Summary of test statistics' *p*‐values of Wilcoxon rank sum test, Kruskal–Wallis rank sum test (Rank sum tests of SBL) and Levene's test of variance (Variability of SBL) for standardised body lengths in *Harpalus affinis* and *H. rufipes* from agricultural and urban habitats, rural and city areas across space and time per sex For more detailed information see Tables A1 and A2.

Response variable	Sex	*H. affinis*		*H. rufipes*	
		Rank sum tests SBL	Variability of SBL	Rank sum tests SBL	Variability of SBL
Sex (female; male)	–	<.0001	–	<.0001	–
Habitat (agricultural; urban)	Female	.728	.225	.142	.598
	Male	.229	.201	.081	.025
Area (rural; city)	Female	.780	.249	.196	.249
	Male	.313	.170	.587	.025
Rural over time	Female	.848	.439	.370	.286
	Male	.586	.615	.315	.584
City over time	Female	.365	.762	.681	.363
	Male	.565	.020	.005	.665

In *H. affinis*, no spatial differences in size variability were found (Tables 2 and A2). However, variability in *H. affinis* males differed between time periods in city areas (*n* = 68; *p* = .020; Tables 2 and A2; Figure 3d). Male size variability was equal in 1892–1949 and 1957–1998 (*n* = 163; *p* unadjusted = .366; *p* = 1), and between 1957–1998 and 2016–2017 (*n* = 135; *p* unadjusted = .026; p = .079) but in 2016–2017 size variability significantly increased compared to 1892–1949 (*n* = 68; *p* unadjusted = .013; *p* = .038; Tables 2 and A2; Figure 3d).

In *H. rufipes* males, body size changed through time in city areas (*n* = 41; *p* < .01; Tables 2 and A1; Figure 4d). Male *H. rufipes* were equally large in 1892–1949 and 1957–1998 (*n* = 29; *p* unadjusted = .350; *p* = 1), but in 2016–2017 males were significantly smaller than in previous time‐periods (1957–1998: *n* = 20; *z* = 2.620; *p* unadjusted = .009; *p* = .026; 1892–1949: *n* = 31; *z* = 3.022; *p* unadjusted = .003; *p* = .008). The variability of body size in both sexes of *H. rufipes* did not differ significantly in rural areas over time. Furthermore, body size variability of *H. rufipes* males in city areas over time did not differ significantly. The variability of body sizes of *H. rufipes* males was, however, significantly higher in urban compared to agricultural habitats (*n* = 45; *p* = .025; Tables 2 and A2; Figure 4a), and also significantly higher in city areas in comparison with rural areas (*n* = 96; *p* = .025; Tables 2 and A2; Figure 4b).

### 3.2 Stable isotopes

The stable nitrogen and carbon isotope composition within particular tissues and body components of both species did not differ between sexes (Tables 3 and A3). For subsequent analyses, we thus pooled the data of males and females of each species.

**TABLE 3:** Summary of test statistics' *p*‐values of Wilcoxon rank sum test, Kruskal–Wallis rank sum test (Rank sum tests stable isotopes), and Levene's test of variance (Variability of stable isotopes) for stable nitrogen (^15^N/^14^N) and carbon (^13^C/^12^C) isotope signatures (in ‰) of *Harpalus affinis* and *H. rufipes* tissues and body components (cuticula, legs, muscles) from agricultural and urban habitats. For more detailed information see Tables A3 and A5.

Stable isotopes	Tissues	Response variable	*H. affinis*		*H. rufipes*	
			Rank sum tests stable isotopes	Variability of stable isotopes	Rank sum tests of stable isotopes	Variability of stable isotopes
^15^N/^14^N	Cuticula	Sex (female; male)	.248	–	.739	–
	Legs		.353	–	.684	–
	Muscles		.622	–	.971	–
	Cuticula	Habitat (agricultural; urban)	.007	.851	.023	.207
	Legs		.002	.884	.043	.058
	Muscles		.093	.478	.007	.011
^13^C/^12^C	Cuticula	Sex (female; male)	.496	–	.436	–
	Legs		.796	–	.315	–
	Muscles		1.000	–	.529	–
	Cuticula	Habitat (agricultural; urban)	.496	.827	.393	.908
	Legs		.971	.833	.315	.573
	Muscles		.833	.428	.190	.748

**FIGURE 5:**
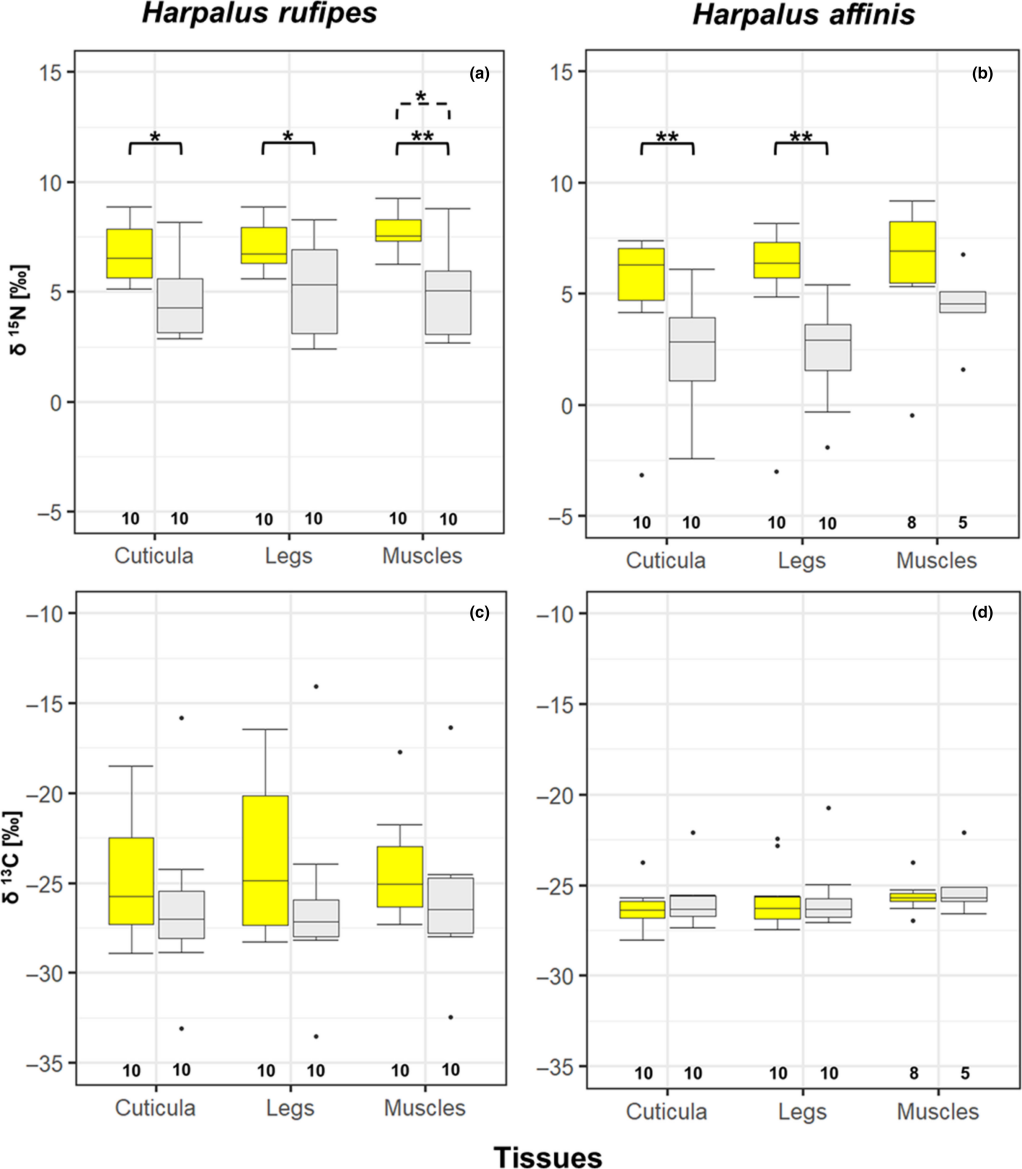
Stable δ^15^N and δ^13^C (in ‰) in cuticula, legs, and muscles of *Harpalus rufipes* (a; c) and *H. affinis* (b; d) from agricultural (yellow boxes) and urban (grey boxes) habitats, boxes mark the interquartile ranges, whiskers indicate the minimum and maximum values, horizontal lines in boxes indicate medians, dots above and below boxes represent outliers, numbers below box plots provide sample sizes, black brackets with stars indicate significant difference between stable isotope values in tissues/body components between habitats, dotted bracket with stars indicate significant differences in variability of stable isotope values in tissues between habitats (*p < .05; **p < .01).

^15^N values were significantly higher in cuticula and legs of *H. affinis* from agricultural habitats compared to urban habitats (cuticula: *n* = 10, *p* ≤ .01; legs: *n* = 10; *p* ≤ .01; Tables 3 and A3; Figure 5b). The nitrogen isotope signature of *H. affinis* muscles did not significantly differ between habitats (Tables 3 and A3; Figure 5b). We found no differences in the variability of δ ^15^N values in different tissues or body components of *H. affinis*, originating from agricultural or urban habitats. ^15^N was significantly higher in the cuticula, leg and muscles tissues of *H. rufipes* beetles originating from agricultural habitats than those from urban habitats (cuticula: *n* = 10, *p* > .05; legs: *n* = 10; *p* > .050; muscles: *n* = 10, *p* > .01; Tables 3 and A3; Figure 5a). Variability of δ ^15^N values of *H. rufipes* muscles from urban habitats was significantly higher than in agricultural habitats (muscles: *n* = 20; *p* = .011; Table A5; Figure 5a).

We detected no significant differences in carbon isotope signature values or value variability in tissues/body components of *H. affinis* and *H. rufipes* related to different habitats (Tables 3 and A3; Figure 5c,d). The comparison of nitrogen and carbon stable isotope signatures between cuticula, legs and muscles of specimens of both species either from agricultural or from urban habitats yielded no significant differences either (Table A4).

## 4 DISCUSSION

Intraspecific morphological and biochemical trait changes, caused by human‐induced environmental changes, may occur across space and time (Doudna & Danielson, 2015; Keinath et al., 2020, 2021; Niemeier et al., 2020; Van't Hof et al., 2011) and might enable species to persist in changing habitats. Our study focussed on body size and isotopic signatures of two ground beetle species, *Harpalus affinis* and *H. rufipes*. Both species persisted in rural and urban areas of the Berlin‐Brandenburg region across the past 125 years, despite profound environmental changes. In our study we predicted specimens of both species to decrease in their body size from the past to the present in the city (Sukhodolskaya, 2013; Weller & Ganzhorn, 2003), reflecting habitat conditions and increasing urban temperatures (Fenner et al., 2018; Gillooly et al., 2001; Kingsolver & Huey, 2008; Li et al., 2018). Across a spatial gradient from rural to urban habitats, we assumed stable nitrogen and carbon enrichments to be reflected in specimens' tissues and body compounds when occurring in differently human‐induced habitats. We detected some intraspecific morphological trait changes to occur across 125 years, depending on the respective species, activity pattern and sexes, as well as biochemical trait changes between populations occurring in differently human impacted habitats.

### 4.1 Body size in carabids over time

We found no spatio‐temporal changes in body size in both sexes of *H. affinis* and *H. rufipes'* females. However, we detected a decrease in *H. rufipes* males' body size in the city across 125 years. Males of *H. rufipes* from city areas were significantly smaller recently (2016–2017) compared to males from former time periods (1892–1949; 1957–1998).

In many taxa, intraspecific variation in body size is known to be influenced by environmental temperatures, primarily in ectotherms, that is amphibians (Reading, 2007), fish (Todd et al., 2008) and insects (Kingsolver & Huey, 2008). In insects, occurring in habitats with limited food sources, higher environmental temperatures lead to an earlier start of metamorphosis, resulting in smaller‐sized imagines (Gillooly et al., 2001; Kingsolver & Huey, 2008). Because the predominant food source in both studied ground beetle species are weed seeds (Bažok et al., 2007; Luff, 2002; Sunderland et al., 1995; Toft & Bilde, 2002; Townsend, 1992; Trautner, 2017), we assumed urban habitats, in addition to their higher temperatures, to be lower in habitat quality than rural areas with a high amount of agricultural landscapes. Berlin exhibits higher temperatures than its rural surroundings (Li et al., 2018), and its temperature increased with increasing urbanisation over time (Fenner et al., 2018; Tseng et al., 2018). Thus, higher and/or increasing temperatures within the city were assumed to result in smaller and/or decreasing body size in both species, as described for other arthropods (Brans et al., 2017; Kotze et al., 2011).

However, because only *H. rufipes* males' body size decreased in the city over time, we concluded that insufficient habitat quality in combination with urban heat was not the main driver for these changes. Because *H. affinis* and *H. rufipes* both have the same food source, insufficient habitat quality and higher temperatures in the city should also have an effect on body size in *H. rufipes* females and both sexes of *H. affinis*. However, there might be reasons female size is more constrained than males, based on ability to produce eggs and/or behavioural differences (Thornhill & Alcock, 1983). In addition, we did not detect any spatial intraspecific differences in body size between habitats (agricultural, urban) and areas (rural, city) in both species. Thus, we refute our hypotheses that insufficient habitat quality in the city and increasing urban heat lead to smaller‐sized beetles compared to rural areas in space and time.

The reason for species‐ and sex‐specific decrease in body size over time might be caused by other urban environmental conditions that changed over time. Therefore, differences in the biology between *H. affinis* and *H. rufipes*, as well as between *H. rufipes* sexes have to be considered. Both species differ in their activity pattern. Whereas *H. affinis* is diurnal, *H. rufipes* is predominantly nocturnal (Wrase, 2004).

The sexes of *H. rufipes* differ regarding their dispersal activities, showing that males have better developed flight muscles than females, and are more active via flying when searching for mates (Matalin, 2003). Among insects, males generally expend most of their reproductive energy in mating effort, that is movement to encounter mates, while females expend more energy in egg production (Thornhill & Alcock, 1983).

Because of *H. rufipes*' nocturnal activity, this species is known to be attracted to artificial nightlights (Kegel, 1990; Matalin, 2003; Szentkirályi et al., 2003). Potentially, male *H. rufipes*, searching for females in the city nocturnally, might be distracted and attracted by streetlights, captured in its light beam, unable to escape and die due to predation or exhaustion, as was shown for other nocturnal insects (Eisenbeis, 2006; Manfrin et al., 2018; Rydell, 1992). A study by Lagisz (2008) showed that relatively larger wing size in *Pterostichus oblongopunctatus* (Fabricius, 1787) is related to better dispersal abilities. Matalin (2003) examined in different ground beetle species, including *H. rufipes*, relative wing surface to be an indicator for dispersal abilities. Thus, larger specimens might have better dispersal abilities than smaller ones. Hence, larger *H. rufipes* males might have better flight capacities to disperse over longer distances when searching for females and might therefore be more disturbed by street lights than smaller ones. Although larger individuals are better dispersers, this may come with the cost of lower reproductive rates (Crawley, 1989). Thus, increasing the number of streetlights in Berlin across the past 150 years (Eisenbeis & Hänel, 2009; Keinath et al., 2021; Kyba et al., 2017) might have led to higher selection pressures on large‐sized males and resulted in an adaptive advantage for smaller‐sized males in the city.

By contrast, body sizes of *H. rufipes* males remained nearly constant in rural areas over the investigated timeframe. Rural environments are less impacted by street lights (Rich & Longcore, 2006) and should have little impact on *H. rufipes* males. However, *H. rufipes* males' body size showed high variation in urban habitats. This might be explained by the heterogeneous habitat structure in cities, because habitats with less or no street lights can even exist within cities (Rich & Longcore, 2006). Likewise, the body size in males of the diurnal *H. affinis* showed larger variability in the city in recent times (2016–2017) compared with the past (1892–1949). This finding might be also reasoned by higher habitat heterogeneity in the city, ranging from highly urbanised to almost natural areas, making Berlin to one of the greenest but simultaneously highly populated European cities (Schewenius et al., 2014).

The hypothetical explanation based on activity during the day and sex‐dependent dispersal abilities would explain why both sexes of the diurnal *H. affinis* and the less mobile females of the nocturnal *H. rufipes* showed no changes in body size through time and that morphological trait changes might be dependent on the respective activity pattern of a species, and different behaviours of their sexes. In the diurnal *H. affinis*, sex‐specific adaptation in coloration to urbanisation across the same timeframe was found (Keinath et al., 2020).

### 4.2 Spatial nitrogen enrichments in carabids

We detected higher nitrogen isotopic enrichments in individuals of both species from landscapes with intensive agriculture compared to urban habitats. In particular, we found higher nitrogen isotopic signatures in all tissues and body components representing nutritive signatures of the larval stages (Gratton & Forbes, 2006; Peterson & Fry, 1987). Stable nitrogen enrichments might be higher in individuals because of their position in the food web, or when the respective environment is highly enriched by nitrogen, such as in agricultural landscapes due to fertiliser use (Birkhofer et al., 2011). Because both ground beetle species are feeding predominantly on weed seeds (Freyer & Aly, 1974; Jenkinson, 2001; Shearer et al., 1974), our results indicate that larvae occurring in agricultural habitats might have fed on highly nitrogenic enriched weed seeds, what might be caused by fertiliser use. This was also described by Birkhofer et al. (2011) for other arthropods, showing that nitrogen isotopic compositions are increasing with the intensity level of agricultural land usage. However, in *H. affinis*' tissues, representing adult stage, we detected no difference in the stable nitrogen isotope composition between specimens from agricultural and urban habitats, suggesting that *H. affinis* did not disperse into isotopically distinct habitats after larval development. This particularly also seems to apply to *H. affinis* adults from urban habitats seemingly living in habitats with nitrogen isotope ratios comparable to those of intense agricultural ones or feeding on different diets compared to rural beetles.

The assumption that some urban habitats might be similarly high enriched by nitrogen as agricultural ones, may again be underlined by the higher variability of nitrogen isotope values in *H. rufipes* tissues, representing adult stages, in urban habitats compared to agricultural ones. Agricultural habitats are more homogenous, in particular due to the presence of monocultures (Jongman, 2002), whereas urban environments exhibit more heterogeneous structures such as parks, private gardens, ruderal areas and green strips (Falk, 1976; Gill & Bonnett, 1973; Tischler, 1973). Thus, urban habitats are also variable in their nitrogen enrichments (Pyšek, 1995), depending on the degree of anthropogenic impact, that is the proximity of streets due to automobile exhaust (Baker et al., 2001), the use of fertiliser for maintaining lawns (Muchovej & Rechcigl, 1994), the ‘structure’ of the city (amount and pattern of green versus built cover space), and the deposit of excretions of pets (Zhu et al., 2004). Increased nitrogen isotopic variability was also found in the European Common Frog, *Rana temporaria*, when occurring in non‐agricultural environments in comparison with agricultural landscapes within the Berlin‐Brandenburg region (Niemeier et al., 2020).

### 4.3 Spatial carbon enrichments in carabids

We found no significant differences in carbon signatures in both species' tissues and body components between agricultural and urban habitats. However, in *H. rufipes* higher carbon isotope signatures were visible when sampled from agricultural habitats. Although this effect was not significant, it might be explained by the higher proportion of C4 plants, like corn, in arable fields than in the city (Degens, 1969; Schwarcz, 1969).

## 5 CONCLUSIONS

Our results revealed spatio‐temporal intraspecific morphological and biochemical trait changes in response to human‐induced environmental alterations. Morphological changes occurred across the relative short timeframe of 125 years in our study area, depend on the respective species, their sex and activity pattern. Furthermore, we found nitrogen enrichments in the environment to influence biochemical traits in specimens, occurring in habitats that are differently human‐induced.

## AUTHOR CONTRIBUTIONS

**Silvia Keinath:** Conceptualization (equal); formal analysis (lead); investigation (lead); visualization (lead); writing – original draft (lead); writing – review and editing (lead). **Johannes Frisch:** Conceptualization (equal); resources (lead); validation (equal); writing – original draft (supporting); writing – review and editing (supporting). **Johannes Müller:** Conceptualization (equal); funding acquisition (equal); project administration (equal); validation (equal); writing – original draft (supporting); writing – review and editing (supporting). **Frieder Mayer:** Conceptualization (equal); funding acquisition (equal); project administration (equal); validation (equal); writing – original draft (supporting); writing – review and editing (supporting). **Ulrich Struck:** Data curation (equal); methodology (equal); resources (equal); validation (equal); writing – original draft (supporting); writing – review and editing (supporting). **Mark‐Oliver Rödel:** Conceptualization (lead); data curation (equal); funding acquisition (equal); project administration (equal); supervision (lead); validation (lead); writing – original draft (supporting); writing – review and editing (supporting).

## CONFLICT OF INTEREST STATEMENT

We declare no conflicts of interest.

## FUNDING INFORMATION

This work was funded by the German Federal Ministry of Education and Research (BMBF) within the Collaborative Project 'Bridging in Biodiversity Science ‐ BIBS' (funding number: 16LC1501F1).

## Data Availability

Sampling locations, sampling years, habitat types, morphological data and stable nitrogen and carbon data: Database of the Museum für Naturkunde, Berlin https://doi.org/10.7479/stwb‐nf68.

## APPENDIX 1

**TABLE A1:** Body lengths (in mm) of *Harpalus affinis* and *H. rufipes* from agricultural and urban habitats, rural and city areas across space and time per sex.

Species	Comparison	Sex	Medians [mm]		Test	*w*‐Value	Chi‐square	*p*‐Value
*H. affinis*	SBL ~ Sex	–	Female	9.01	Wilcox.	63,318	–	<.**0001**
			Male	8.70				
	SBL ~ Habitat	Female	Agricultural	9.22	Wilcox.	78.5	–	.728
			Urban	8.88				
		Male	Agricultural	8.51		170.5	–	.229
			Urban	8.70				
	SBL ~ Area	Female	Rural	9.07	Wilcox.	7468.5	–	.780
			City	9.01				
		Male	Rural	8.76		19,296	–	.313
			City	8.63				
	SBL ~ Rural over time	Female	1892–1949	8.88	Krusk.–Wal.	–	0.3305	.848
			1957–1998	9.07				
			2016–2017	9.22				
		Male	1892–1949	8.76		–	1.0705	.586
			1957–1998	8.76				
			2016–2017	8.51				
	SBL ~ City over time	Female	1892–1949	9.09	Krusk.–Wal.	–	2.0146	.365
			1957–1998	9.01				
			2016–2017	8.88				
		Male	1892–1949	8.62		–	1.1425	.565
			1957–1998	8.63				
			2016–2017	8.70				
*H. rufipes*	SBL ~ Sex	–	Female	13.46	Wilcox.	6557	–	<.**0001**
			Male	12.53				
	SBL ~ Habitat	Female	Agricultural	13.52	Wilcox.	272	–	.142
			Urban	13.17				
		Male	Agricultural	12.64		298	–	.081
			Urban	12.15				
	SBL ~ Area	Female	Rural	13.52	Wilcox.	775	–	.196
			City	13.17				
		Male	Rural	12.52		1046.5	–	.587
			City	12.57				
	SBL ~ Rural over time	Female	1892–1949	13.22	Krusk.–Wal.	–	1.9911	.370
			1957–1998	13.64				
			2016–2017	13.52				
		Male	1892–1949	12.38		–	2.3087	.315
			1957–1998	12.29				
			2016–2017	12.64				
	SBL ~ City over time	Female	1892–1949	12.96	Krusk.–Wal.	–	0.76896	.681
			1957–1998	13.64				
			2016–2017	12.87				
		Male	1892–1949	12.93		–	10.516	.**005**
			1957–1998	13.09				
			2016–2017	11.76				

*Note*: Results of Wilcoxon rank sum tests (Wilcox.) for comparisons of body lengths between females and males, for comparisons of body lengths between habitats in females and males, and for comparisons of body lengths between areas in females and males. Results of Kruskal–Wallis rank sum tests (Krusk.–Wal.) for comparisons of body lengths between time periods in rural areas in females and males, and between time periods in city areas in females and males, significant differences are given in bold.

**TABLE A2:** Body lengths (in mm) of *Harpalus affinis* and *H. rufipes* from agricultural and urban habitats, rural and city areas across space and time per sex.

Species	Comparison	Sex	Medians		df	*F*‐Value	*p*‐Value
*H. affinis*	SBL ~ Habitat	Female	Agricultural	0.357	22	1.559	.225
			Urban	0.177			
		Male	Agricultural	0.268	40	1.694	.201
			Urban	1.212			
	SBL ~ Area	Female	Rural	0.399	240	1.538	.249
			City	0.355			
		Male	Rural	0.251	380	1.887	.170
			City	0.444			
	SBL ~ Rural over time	Female	1892–1949	0.5745	121	0.829	.439
			1957–1998	0.322			
			2016–2017	0.357			
		Male	1892–1949	0.189	196	0.488	.615
			1957–1998	0.275			
			2016–2017	0.268			
	SBL ~ City over time	Female	1892–1949	0.336	115	0.273	.762
			1957–1998	0.389			
			2016–2017	0.195			
		Male	1892–1949	0.192	180	4.010	.**020**
			1957–1998	0.394			
			2016–2017	1.368			
*H. rufipes*	SBL ~ Habitat	Female	Agricultural	0.366	53	0.281	.598
			Urban	0.229			
		Male	Agricultural	0.364	43	5.391	.**025**
			Urban	0.884			
	SBL ~ Area	Female	Rural	0.399	240	1.338	.249
			City	0.355			
		Male	Rural	0.434	94	5.157	.**025**
			City	0.764			
	SBL ~ Rural over time	Female	1892–1949	0.720	59	1.279	.286
			1957–1998	0.882			
			2016–2017	0.366			
		Male	1892–1949	0.525	52	0.543	.584
			1957–1998	0.526			
			2016–2017	0.364			
	SBL ~ City over time	Female	1892–1949	0.664	18	1.072	.363
			1957–1998	3.196			
			2016–2017	0.269			
		Male	1892–1949	0.635	37	0.413	.665
			1957–1998	0.317			
			2016–2017	0.662			

*Note*: Results of Levene's test for homogeneity of variance, centre = median. Significant *p*‐values are given in bold.

**TABLE A3:** Stable ^14^N and ^13^C isotope enrichments (in %) in cuticula, legs, and muscles of *Harpalus affinis* and *H. rufipes* between sexes and specimens from agricultural and urban habitats.

Species	Tissue	Comparison	Medians		*w*‐Value	*p*‐Value
*H. affinis*	Cuticula	δ^15^N ~ Sex	Female	5.816	66	.248
			Male	3.082		
		δ^13^C ~ Sex	Female	−26.656	40.5	.496
			Male	−26.134		
		δ^15^N ~ Habitat	Agricultural	6.283	85	.**007**
			Urban	2.839		
		δ^13^C ~ Habitat	Agricultural	−26.389	40.5	.496
			Urban	−26.311		
	Legs	δ^15^N ~ Sex	Female	5.601	63	.353
			Male	3.177		
		δ^13^C ~ Sex	Female	−26.426	46	.796
			Male	−26.289		
		δ^15^N ~ Habitat	Agriculture	6.3840	89	.**002**
			Urban	2.9185		
		δ^13^C ~ Habitat	Agricultural	−26.301	49	.971
			Urban	−26.313		
	Muscles	δ^15^N ~ Sex	Female	6.754	24	.622
			Male	5.310		
		δ^13^C ~ Sex	Female	−25.662	20	1.000
			Male	−25.705		
		δ^15^N ~ Habitat	Agricultural	6.9245	32	.093
			Urban	4.534		
		δ^13^C ~ Habitat	Agricultural	−25.693	18	.833
			Urban	−25.686		
*H. rufipes*	Cuticula	δ^15^N ~ Sex	Female	6.287	55	.739
			Male	5.495		
		δ^13^C ~ Sex	Female	−25.755	61	.436
			Male	−27.053		
		δ^15^N ~ Habitat	Agricultural	6.540	80	.**023**
			Urban	4.273		
		δ^13^C ~ Habitat	Agricultural	−25.755	62	.393
			Urban	−27.019		
	Legs	δ^15^N ~ Sex	Female	6.307	44	.684
			Male	6.344		
		δ^13^C ~ Sex	Female	−24.870	64	.315
			Male	−27.161		
		δ^15^N ~ Habitat	Agricultural	6.718	77	.**043**
			Urban	5.304		
		δ^13^C ~ Habitat	Agricultural	−24.870	64	.315
			Urban	−27.144		
	Muscles	δ^15^N ~ Sex	Female	7.363	51	.971
			Male	6.749		
		δ^13^C ~ Sex	Female	−25.088	59	.529
			Male	−26.320		
		δ^15^N ~ Habitat	Agricultural	7.557	85	.**007**
			Urban	5.053		
		δ^13^C ~ Habitat	Agricultural	−25.088	68	.190
			Urban	−26.491		

*Note*: Results of Wilcoxon rank sum tests for comparisons between tissues of females and males and between beetles originated from agricultural and urban habitats. Significant differences are given in bold.

**TABLE A4:** Stable ^14^N and ^13^C isotope enrichments between cuticula, legs, and muscles of *Harpalus affinis* and *H. rufipes* from agricultural and urban habitats.

Species	Habitat	Comparison	Tissue	Medians	Chi‐squared	df	*p*‐Value
*H. affinis*	Agricultural	δ^13^C ~ Tissue	Cuticula	−26.3885	2.2999	2	.317
			Legs	−26.3005			
			Muscles	−25.6925			
		δ^15^N ~ Tissue	Cuticula	6.2830	1.2739	2	.529
			Legs	6.3840			
			Muscles	6.9245			
	Urban	δ^13^C ~ Tissue	Cuticula	−26.3105	1.5102	2	.470
			Legs	−26.3130			
			Muscles	−25.6860			
		δ^15^N ~ Tissue	Cuticula	2.8390	3.3729	2	.185
			Legs	2.9185			
			Muscles	4.5340			
*H. rufipes*	Agricultural	δ^13^C ~ Tissue	Cuticula	−25.7545	0.34323	2	.8423
			Legs	−24.8695			
			Muscles	−25.0880			
		δ^15^N ~ Tissue	Cuticula	6.5395	2.6039	2	.272
			Legs	6.7180			
			Muscles	7.5579			
	Urban	δ^13^C ~ Tissue	Cuticula	−27.019	0.63304	2	.7287
			Legs	−27.144			
			Muscles	−26.491			
		δ^15^N ~ Tissue	Cuticula	4.2730	0.018065	2	.991
			Legs	5.3040			
			Muscles	5.0525			

*Note*: Results of Kruskal–Wallis rank sum tests for comparisons between tissues of beetles originated from agricultural and urban habitats.

**TABLE A5:** Stable ^15^N and ^13^C isotope enrichments (in ‰) in cuticula, legs, and muscles of *Harpalus affinis* and *H. rufipes* from agricultural and urban habitats.

Species	Tissue	Comparison	Medians		df	*F*‐Value	*p*‐Value
*H. affinis*	Cuticula	δ^15^N ~ Habitat	Agricultural	9.944	18	0.0362	.851
			Urban	7.225			
		δ^13^C ~ Habitat	Agricultural	1.240	18	0.049	.827
			Urban	2.114			
	Legs	δ^15^N ~ Habitat	Agricultural	10.163	18	0.0221	.884
			Urban	4.720			
		δ^13^C ~ Habitat	Agricultural	3.017	18	0.0457	.833
			Urban	3.528			
	Muscles	δ^15^N ~ Habitat	Agricultural	9.262	18	0.5384	.478
			Urban	3.472			
		δ^13^C ~ Habitat	Agricultural	0.835	18	0.6774	.428
			Urban	3.078			
*H. rufipes*	Cuticula	δ^15^N ~ Habitat	Agricultural	1.877	18	1.7107	.207
			Urban	3.894			
		δ^13^C ~ Habitat	Agricultural	13.396	18	0.0137	.908
			Urban	19.419			
	Legs	δ^15^N ~ Habitat	Agricultural	1.255	18	4.0902	.058
			Urban	4.932			
		δ^13^C ~ Habitat	Agricultural	18.588	18	0.3294	.573
			Urban	24.233			
	Muscles	δ^15^N ~ Habitat	Agricultural	0.778	18	7.9687	.**011**
			Urban	4.713			
		δ^13^C ~ Habitat	Agricultural	8.678	18	0.1067	.748
			Urban	16.596			

*Note*: Results of Levene's Test for Homogeneity of Variance, centre = median. Significant *p*‐values are given in bold.
